# Circulating tRF-Gly-GCC as a biomarker for colorectal cancer and Crohn’s disease activity

**DOI:** 10.1038/s41598-026-59591-8

**Published:** 2026-06-30

**Authors:** Sarah Salah, Fatma Dwedar, Rasha Nassra, Abeer Mahmoud, Mai Zahra

**Affiliations:** 1 Department of Medical Biochemistry, Faculty of Medicine, Alexandria University, Alexandria, Egypt; 2 Department of Internal Medicine, Faculty of Medicine, Alexandria University, Alexandria, Egypt

**Keywords:** Crohn’s disease, Colorectal cancer, tRF-Gly-GCC, tRNA-derived fragments, Disease activity, Biomarker, Biomarkers, Cancer, Gastroenterology

## Abstract

Distinguishing intestinal inflammation from colorectal cancer remains challenging using non-invasive biomarkers. tRNA-derived fragments have been implicated in cancer, but their role in Crohn’s disease activity and their ability to differentiate inflammatory from malignant intestinal conditions remain unclear. This study evaluated circulating tRF-Gly-GCC in patients with active Crohn’s disease (*n* = 20), inactive Crohn’s disease (*n* = 20), colorectal cancer (*n* = 24), and healthy controls (*n* = 21). Serum tRF-Gly-GCC expression levels showed a directional increase across disease states, from controls to inactive Crohn’s disease, active disease, and colorectal cancer. A Jonckheere–Terpstra test formally confirmed a significant monotonic increase (z = 7.896, *p* < 0.001). The marker distinguished colorectal cancer from controls with high sensitivity (95.8%) and specificity (95.2%), and differentiated colorectal cancer from Crohn’s disease with sensitivity of 95.8% and specificity of 80.0%. It also distinguished active from inactive Crohn’s disease with 90% sensitivity and 90% specificity. In Crohn’s disease, tRF-Gly-GCC correlated with disease activity indices but not with fecal calprotectin. These findings suggest that circulating tRF-Gly-GCC reflects intestinal inflammatory activity and may serve as a non-invasive biomarker for disease assessment and differentiation between inflammatory and malignant conditions.

**Supplementary Information:**

The online version contains supplementary material available at 10.1038/s41598-026-59591-8.

## Introduction

Nearly one in four human cancers arises from chronic infection or inflammation^1^. The connection between chronic inflammation and cancer dates back to the 19th century, when Rudolf Virchow observed leukocyte infiltration in tumors. He hypothesized that ongoing inflammation could promote malignant transformation^2^. Modern research confirms that persistent inflammation damages epithelial integrity, induces DNA mutations, and causes genomic instability. These changes create an environment favorable for cancer development^3–6^. Well-established examples include Helicobacter pylori infection in gastric cancer, viral hepatitis-related hepatocellular carcinoma, and pancreatitis-associated pancreatic cancer^3,5,6^. In colorectal cancer (CRC), both sporadic immune dysregulation and inflammatory bowel disease (IBD) act as important drivers of tumorigenesis^4,7,8^.

In IBD, ulcerative colitis has historically received greater attention as a risk factor for colorectal cancer, while cancer risk in Crohn’s disease has been comparatively underemphasized. Nevertheless, patients with colonic Crohn’s disease also face a substantial risk of colorectal cancer^9–11^, with population-based studies demonstrating an approximately twofold increase compared with the general population^12^. Differentiating colorectal cancer from inflammatory disease in Crohn’s colitis remains particularly challenging. The segmental and transmural nature of Crohn’s inflammation, together with the frequent presence of strictures and fistulas, may obscure malignant lesions and complicate histological sampling^13,14^. In addition, active Crohn’s disease and colorectal cancer share overlapping clinical features, including abdominal pain, diarrhea, anemia, and weight loss, which may delay diagnosis or lead to misinterpretation of symptoms^15,16^. Chronic inflammation further induces structural bowel alterations that limit the diagnostic accuracy of endoscopic and radiologic assessment^16^.

Serum tumor markers such as carcinoembryonic antigen (CEA) and carbohydrate antigen 19 − 9 (CA19-9) are commonly utilized in routine clinical practice to support the diagnosis and monitoring of colorectal cancer. However, their clinical utility is restricted by limited sensitivity in early-stage disease and decreased specificity during active intestinal inflammation. These limitations present difficulties in distinguishing inflammatory activity in Crohn’s disease from colorectal malignancy, particularly among patients with long-standing colonic involvement^17,18^.

Beyond cancer detection, accurate evaluation of inflammatory burden in Crohn’s disease also remains difficult. Current clinical practice relies on patient-based tools like the Crohn’s Disease Activity Index (CDAI) and endoscopic assessment methods including the Simple Endoscopic Score for Crohn’s Disease (SES-CD). While endoscopy remains the reference standard for assessing mucosal activity, its invasiveness and limited feasibility for repeated monitoring restrict routine use^19^. Clinical indices and endoscopic scores often correlate poorly with one another and do not fully capture mucosal or molecular inflammatory activity, resulting in frequent discordance between symptoms, endoscopic findings, and the underlying disease biology^20–22^. Non-invasive biomarkers such as fecal calprotectin are widely applied but demonstrate variable correlation with endoscopic severity in Crohn’s disease, particularly in patients with isolated small bowel involvement or stricturing disease^23,24^.

Despite these diagnostic challenges, current surveillance strategies continue to focus predominantly on ulcerative colitis, leaving Crohn’s disease-associated colorectal cancer relatively understudied and potentially under-monitored^10,25,26^. Together, these limitations underscore the need for biomarkers that can both aid in differentiating colorectal cancer from Crohn’s disease and provide reliable assessment of inflammatory activity. These shortcomings have driven interest in circulating molecular biomarkers that better reflect disease-related regulatory and inflammatory processes.

Recent advances in transcriptomics have transformed our understanding of gene regulation. Over 90% of the human genome is transcribed into non-coding RNAs (ncRNAs), which, despite not encoding proteins, regulate gene expression, chromatin structure, and cell fate decisions^27–29^. Among ncRNAs, tRNA-derived fragments (tRFs) have emerged as important small noncoding RNAs (14–50 nt) generated by precise cleavage of mature or precursor tRNAs. They regulate gene expression at transcriptional and post-transcriptional levels, including gene silencing, RNA processing, and translation, and play key roles in stress responses, immune signaling, and cancer biology. Aberrant tRF expression is associated with diseases such as cancer, neurodegeneration, viral infection, metabolic abnormalities, and inflammation, highlighting their diagnostic, prognostic, and therapeutic potential^30–32^.

One tRF in particular, tRF-Gly-GCC, has emerged as one of the most consistently dysregulated tRFs across different disease states. Increased expression has been documented in hepatocellular carcinoma and colorectal cancer, where it correlates with tumor progression and shows promise as a biomarker^33,34^. Elevated Gly-GCC fragments were also detected in acute coronary syndrome, radiation-induced lung injury, and maternal sera from pregnancies affected by congenital heart disease, suggesting roles in stress responses and developmental pathologies^35–37^. Its presence in circulation further supports the promise of 5′-tRF-Gly-GCC as a biomarker for inflammation and malignancy, consistent with its involvement in immune modulation, DNA damage response, and tumor progression^34,36,38^. Overall, these findings suggest that 5′-tRF-Gly-GCC reflects inflammatory and stress-related molecular dysregulation beyond cancer-specific mechanisms alone. However, its circulating expression profile in Crohn’s disease remains unexplored.

We investigated circulating 5′-tRF-Gly-GCC as a potential biomarker to address two clinical challenges: differentiating colorectal cancer from Crohn’s disease and assessing inflammatory activity in Crohn’s disease. Circulating 5′-tRF-Gly-GCC levels were measured in patients with Crohn’s disease, patients with sporadic colorectal cancer, and healthy controls. In the Crohn’s disease group, we evaluated associations between 5′-tRF-Gly-GCC and disease activity markers, including CDAI scores, endoscopic severity (SES-CD), and fecal calprotectin. We aimed to determine whether 5′-tRF-Gly-GCC could differentiate disease states and reflect inflammatory burden in Crohn’s disease.

## Methods

### Study design and participants

This case–control study included 85 subjects referred to the Gastrointestinal Unit, Department of Internal Medicine, Alexandria Main University Hospital. Participants were classified into three groups:

1. Colorectal cancer (CRC) group: twenty-four patients with histopathologically confirmed colorectal adenocarcinoma. Tumor staging was performed according to the Tumor–Node–Metastasis (TNM) classification system^39^.
2. Crohn’s disease (CD) group: forty patients with an established diagnosis of Crohn’s disease, subdivided into:
  Active disease group (*n* = 20)
  Inactive disease group (*n* = 20)
    Disease activity was assessed clinically using the Crohn’s Disease Activity Index (CDAI) and endoscopically using the Simple Endoscopic Score for Crohn’s Disease (SES-CD)^40.^

3. Control group: twenty-one apparently healthy age- and sex-matched individuals with no history of gastrointestinal, inflammatory, or malignant disease.

Post hoc power was estimated using PASS 2020 (NCSS, LLC, Kaysville, UT, USA)^41^ based on the achieved sample of *N* = 85 and a two-sided significance level of α = 0.05. For the primary biomarker, 5′-tRF-Gly-GCC, the observed AUC values ranged from 0.79 to 0.98 across the four pairwise diagnostic comparisons, yielding estimated post-hoc power of 75–95%.

### Exclusion criteria

The following individuals were excluded:

- Pregnant subjects.
- Subjects with concomitant chronic illnesses (e.g., thyroid disorders, chronic kidney disease, autoimmune diseases, hepatic, cardiac, or collagen vascular diseases).
- Individuals with other malignancies.

### Sample collection and laboratory investigations

Venous blood (5 mL) was collected from each participant into plain tubes with gel separators. After clot formation, samples were centrifuged at 1200 × g for 10 min, and serum was aliquoted and stored at − 80 °C until analysis. Fecal calprotectin and serum tumor markers (CEA and CA 19 − 9) were measured^42^.

### RNA extraction and cDNA synthesis^43,44^

Total RNA was extracted from serum using the Qiagen miRNeasy Mini Kit following the manufacturer’s protocol. RNA purity and concentration were measured using a NanoDrop 2000/2000c spectrophotometer (Thermo Scientific, USA), with A260/A280 and A260/A230 ratios between 1.8 and 2.1 considered acceptable.

Complementary DNA (cDNA) was synthesized from 500 ng of total RNA using the RevertAid First Strand cDNA Synthesis Kit (Cat. No. K1622, Thermo Fisher Scientific, USA). Reverse transcription was performed at 25 °C for 5 min, 42 °C for 60 min, and 70 °C for 5 min. cDNA was stored at − 20 °C until further analysis.

### Quantitative real-time PCR (qRT-PCR)^43,44^

Expression of tRF-Gly-GCC was quantified using Maxima SYBR Green qPCR Master Mix (2X) (Thermo Fisher Scientific, Cat. No. K0251) on an Applied Biosystems real-time PCR system, with U6 small nuclear RNA as the endogenous control. Reactions were performed in duplicate, and negative controls were included in each run. Primer sequences used for qRT-PCR are provided in Supplementary Table S1.

### Relative quantification^45^

Relative gene expression levels were calculated using the comparative cycle threshold (2^–ΔΔCT^) method. For each sample ΔCT was calculated by subtracting the CT of the internal control (U6) from the CT of the target gene. The ΔΔCT was then calculated as the difference between the ΔCT of the sample and the mean ΔCT of the healthy control group. Data are expressed as log_2_ fold change (–ΔΔCT) relative to the mean of healthy controls. Positive values indicate upregulation relative to healthy controls; negative values indicate downregulation.

### Statistical analysis^46^

Data were analyzed using SPSS v20.0 (IBM Corp., USA). Normality was assessed with the Shapiro–Wilk test. Categorical variables were expressed as counts and percentages; continuous variables as mean ± SD or median (IQR), as appropriate. Comparisons employed Student’s t-test or ANOVA for parametric data and Mann–Whitney U or Kruskal–Wallis tests for non-parametric data. A Jonckheere-Terpstra test for ordered alternatives was applied to evaluate the pre-specified monotonic trend in tRF-Gly-GCC across the four ordered disease states (healthy controls, inactive CD, active CD, CRC). Spearman correlation between tRF-Gly-GCC and an ordinal disease-severity rank (1 = healthy controls through 4 = CRC) was also computed. Diagnostic accuracy was assessed via pairwise ROC curve analysis between pre-specified group comparisons. Optimal cutoff values were determined using the Youden index. Confidence intervals were calculated for the area under the curve (AUC), sensitivity, and specificity at each cutoff. For clarity of interpretation, sensitivity, specificity, PPV, and NPV are reported only for biomarkers with statistically significant ROC discrimination (*p* < 0.05). To evaluate potential confounding by demographic variables, a general linear model (GLM) was constructed with tRF-Gly-GCC expression as the dependent variable, disease group and sex as fixed factors, and age as a covariate. Two complementary parameterisations were used: (a) an ordinal model with disease stratum coded as a continuous variable (1 = healthy control through 4 = CRC) to estimate a single trend effect; and (b) a dummy-coded categorical model using healthy controls as the reference group to estimate group-specific coefficients. A separate multivariable linear model was fitted within the Crohn’s disease cohort incorporating SES-CD, age, sex, and disease duration as predictors. Significance was set at *p* ≤ 0.05.

## Results

### Baseline demographic and clinical characteristics

Baseline demographic and clinical characteristics are presented in Table 1. As expected, CRC patients were significantly older than the other groups (mean age 55.9 ± 12.0 years vs. 30.8–33.1 years in CD patients and controls; one-way ANOVA F = 43.474, *p* < 0.001). Sex distribution did not differ significantly across groups (χ² = 5.936, *p* = 0.115). CD patients and controls were well-matched for age (*p* = 0.949 for active CD vs. controls; *p* = 0.989 for inactive CD vs. controls). Disease duration did not differ between active and inactive CD patients (Mann–Whitney U = 172.0, *p* = 0.457). (Table 1)

**Table 1:** Baseline demographic and clinical characteristics by study group.

	CRC (*n* = 24)		Active (*n* = 20)		Inactive (*n* = 20)		Control (*n* = 21)		Test of sig.	*p*
	No.	%	No.	%	No.	%	No.	%		
**Sex**										
Male	12	50.0	10	50.0	5	25.0	5	23.8	χ^2^= 5.936	0.115
Female	12	50.0	10	50.0	15	75.0	16	76.2		
**Age (years)**										
Min. – Max.	40.0–75.0		20.0–48.0		18.0–49.0		25.0–40.0		F = 43.474	< 0.001^*^
Mean ± SD.	55.92 ± 11.99		33.10 ± 8.32		30.75 ± 8.06		31.62 ± 4.41			
p_0_	< 0.001^*^		0.949		0.989					
Sig. bet. grps.	p_1_<0.001^*^,p_2_ < 0.001^*^,p_3_ = 0.832									
**Disease duration (years)**										
Min – Max.			1.30–16.70		0.70–13.70				U = 172.0	0.457
Median (IQR)			5.20 (3.30–9.80)		4.95 (2.95–7.25)					

IQR: inter quartile range, SD: standard deviation, χ^2^: Chi square test, U: Mann Whitney test.

F: one way ANOVA test, pairwise comparison bet. each 2 groups was done using post hoc test (Tukey).

p: p value for comparing between the different studied groups.

p_0_: p value for comparing between control and each other groups.

p_1_: p value for comparing between CRC and active.

p_2_: p value for comparing between CRC and inactive.

p_3_: p value for comparing between active and inactive.

*Statistically significant at *p* ≤ 0.05.

### Disease characteristics and activity indices

Among CRC cases, disease staging was evenly split between stages II and III (each 41.7%), with 16.7% at stage I.

Patients with active Crohn’s disease exhibited significantly higher CDAI (median 298.5) and SES-CD (median 18.0) compared with inactive patients (median CDAI 105.0 and SES-CD 1.0, both *p* < 0.001) (Table 2).

### Inflammatory markers

ESR was significantly higher in CRC than in inactive Crohn’s patients and was also elevated in both CRC and active Crohn’s compared to controls. CRP showed a similar trend, but overall, these markers were not very effective at distinguishing between disease states.

### Fecal calprotectin and serum tumor markers

**Fig. 1:**
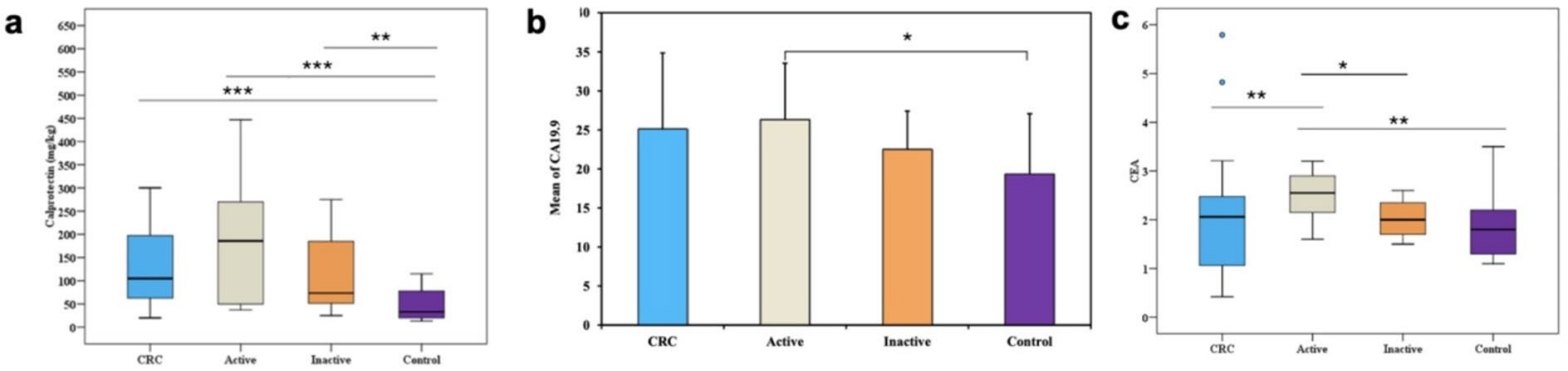
Serum and fecal conventional biomarker levels across study groups. (**a**) Box-and-whisker plot displaying fecal calprotectin levels (mg/kg) in patients with colorectal cancer (CRC; *n* = 24), patients with active Crohn’s disease (*n* = 20), patients with inactive Crohn’s disease (*n* = 20), and healthy controls (*n* = 21). Data are presented as median (interquartile range). Horizontal lines within boxes represent the median; box boundaries represent the 25th and 75th percentiles; whiskers extend to the minimum and maximum values within 1.5× the interquartile range. Overall comparison: Kruskal–Wallis H = 22.529, *p* < 0.001. Pairwise comparisons vs. healthy controls (Dunn’s test): colorectal cancer *p* < 0.001; active Crohn’s disease *p* < 0.001; inactive Crohn’s disease *p* = 0.002. No significant differences were observed between disease groups. (**b**) Bar chart displaying mean serum CA19-9 levels in all study groups. Error bars represent standard deviation. Overall comparison: one-way ANOVA F = 3.357, *p* = 0.023. Pairwise comparison vs. healthy controls (Tukey post-hoc test): active Crohn’s disease *p* = 0.025. No other pairwise comparisons reached statistical significance. **(c)** Box-and-whisker plot displaying serum CEA levels in all study groups. Data are presented as median (interquartile range). Outliers are displayed as individual data points beyond the whiskers. Overall comparison: Kruskal–Wallis H = 11.298, *p* = 0.010. Pairwise comparisons (Dunn’s test): active Crohn’s disease vs. healthy controls *p* = 0.003; colorectal cancer vs. active Crohn’s disease *p* = 0.004; active Crohn’s disease vs. inactive Crohn’s disease *p* = 0.016. No other pairwise comparisons reached statistical significance. Significance thresholds: *p ≤ 0.05; **p ≤ 0.01; ***p < 0.001. CRC, colorectal cancer; CA19-9, carbohydrate antigen 19 − 9; CEA, carcinoembryonic antigen; IQR, interquartile range; SD, standard deviation.

Fecal calprotectin was higher in CRC, active Crohn’s, and inactive Crohn’s compared with controls (*p* < 0.001, *p* < 0.001, and *p* = 0.002, respectively) but did not differ between disease states. CEA and CA19-9 levels differed across the study groups. CEA was significantly higher in active Crohn’s disease compared with colorectal cancer (*p* = 0.004), inactive Crohn’s disease (*p* = 0.016), and healthy controls (*p* = 0.003). No other pairwise comparisons for CEA reached statistical significance. CA19-9 was significantly elevated in active Crohn’s disease relative to healthy controls (*p* = 0.025), whereas differences between the other groups were not statistically significant. (Table 2; Fig. 1)

### Circulating tRF-Gly-GCC expression across study groups

Detectable tRF-Gly-GCC expression was observed across all study groups. Median expression levels showed a directional increase across disease states, with the lowest levels observed in healthy controls, followed by inactive CD, active CD, and the highest levels in CRC. A Jonckheere–Terpstra test for ordered alternatives confirmed a highly significant monotonic increase across the four pre-specified ordered groups (standardized J–T statistic z = 7.896, *p* < 0.001). Spearman correlation between tRF-Gly-GCC and an ordinal disease-severity rank gave r_s_ = 0.793 (*p* < 0.001). (Supplementary Table S2).

**Fig. 2:**
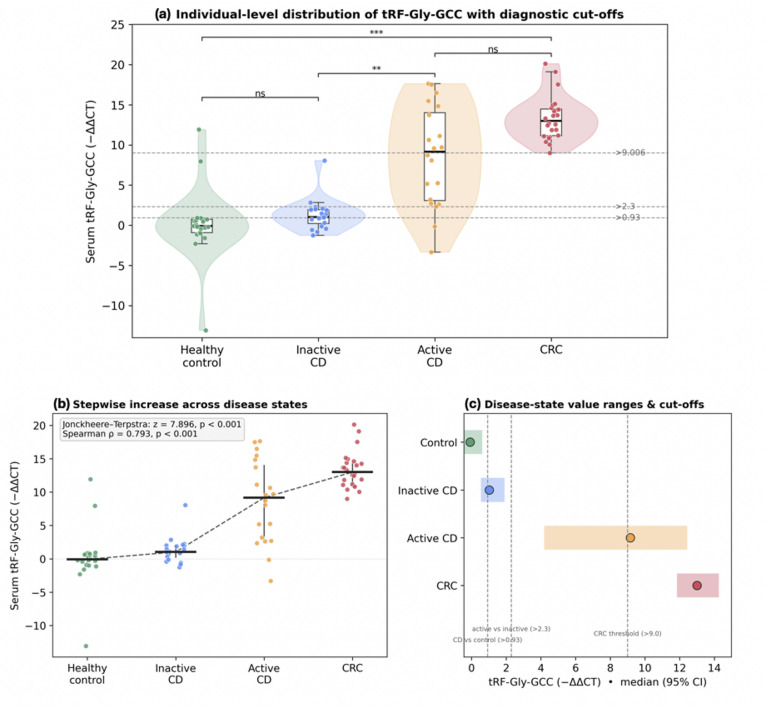
Serum tRF-Gly-GCC relative expression levels across study groups. (**a**) Individual-level distribution with diagnostic cut-offs. Violin plots with superimposed box plots and individual data points (jittered, width = 0.1) displaying the distribution of serum 5’-tRF-Gly-GCC expression (−ΔΔCT) in healthy controls (*n* = 21), inactive Crohn’s disease (*n* = 20), active Crohn’s disease (*n* = 20), and colorectal cancer (*n* = 24). Individual observations are shown to illustrate inter-individual variability and between-group overlap. Horizontal dashed lines indicate the three diagnostic cutoffs derived from pairwise ROC analyses: > 0.93 (optimal cut-off for CD vs. healthy controls), > 2.3 (inactive vs. active CD), > 9.006 (CRC vs. healthy controls). Significance brackets reflect post-hoc pairwise comparisons (Dunn’s test): **p = 0.001; ***p < 0.001; ns, not significant. The comparisons between healthy controls and inactive CD and between active CD and CRC did not reach significance. **(b)** Stepwise increase across disease states. Scatter plot highlighting the upward trend of tRF-Gly-GCC expression evaluated across fundamentally different pathological landscapes. Solid black horizontal lines represent group medians, connected by a dashed trend line. Statistical analyses confirm a highly significant trend across the evaluated cohorts (Jonckheere-Terpstra test: z = 7.896, *p* < 0.001; Spearman’s rank correlation: r_s_ = 0.793, *p* < 0.001). **(c)** Disease-state value ranges and cut-offs. Individual patient tRF-Gly-GCC values shown as dots against group assignment. Coloured horizontal bands represent diagnostic decision zones derived from sequential ROC cutoffs: green zone (≤ 0.93, consistent with healthy control range); blue zone (0.93 to 2.3, inactive CD range); orange zone (2.3 to 9.71, active CD range); red zone (> 9.71, CRC range). The upper zone boundary of 9.71 represents the optimal cut-off for discriminating CRC from the combined CD cohort (all 40 patients); the cut-off of 9.006 shown in panel (a) represents the optimal threshold for discriminating CRC from healthy controls only. Vertical dashed lines map the specific diagnostic thresholds established in panel (a), visually demonstrating how group confidence intervals segregate across these clinical decision boundaries. CRC, colorectal cancer; CD, Crohn’s disease; tRF-Gly-GCC, 5’-tRNA-derived fragment glycine GCC; −ΔΔCT, comparative cycle threshold method for relative gene expression quantification; IQR, interquartile range.

Post hoc pairwise comparisons (Dunn’s test) suggested separation of the cohort into two tiers: a lower-expression/quiescent tier (healthy controls and inactive CD, medians ≈ -0.08 and 1.02) and a higher-expression /active-neoplastic tier (active CD and CRC, medians 9.15 and 13.0). Two adjacent pairwise comparisons were not significant - healthy control vs. inactive CD and active CD vs. CRC. All other comparisons were highly significant (Table 2; Fig. 2).

**Table 2:** Disease activity indices (Section A) and biomarker comparisons across study groups (Section B).

A. Disease activity indices (CD)				
	Active CD (*n* = 20)	Inactive CD (*n* = 20)	Test of sig	*p*
**CDAI**				
Min – max	112.0–482.0	50.0–160.0	U = 12.0^*^	< 0.001^*^
Median (IQR)	298.5 (247.0–381.5)	105.0 (69.50–140.5)		
**SES-CD**				
Min – max	6.0–33.0	0.0–2.0	U = 0.00^*^	< 0.001^*^
Median (IQR)	18.0 (14.50–27.0)	1.0 (0.0–2.0)		

B. Biomarker comparisons across study groups.						
	CRC (*n* = 24)	Active CD (*n* = 20)	Inactive CD (*n* = 20)	Control (*n* = 21)	Test of Sig	p
**Calprotectin (mg/kg)**						
Min – max	20.0–300.0	37.0–447.0	25.0–275.0	13.0–115.0	H= 22.529^*^	< 0.001^*^
Median (IQR)	105.0 (62.50–197.5)	186.0 (50.0–270.0)	73.50 (51.33–185.0)	33.0 (20.0–78.0)		
**vs. Control (p**_**0**_ **)**	< 0.001^*^	< 0.001^*^	0.002^*^			
**Pairwise**	p_1_=0.404,p_2_ = 0.584,p_3_ = 0.186					
**CA19-9 (U/mL)**						
Min – Max	9.25–43.05	12.0–40.0	14.0–31.0	9.0–36.0	F = 3.357^*^	0.023^*^
Mean ± SD.	25.11 ± 9.74	26.31 ± 7.22	22.50 ± 4.91	19.35 ± 7.72		
**vs. Control (p**_**0**_ **)**	0.068	0.025^*^	0.562			
**Pairwise**	p_1_=0.956, p_2_ = 0.680, p_3_ = 0.406					
**CEA (ng/mL)**						
Min. – Max.	0.42–5.79	1.60–3.20	1.50–2.60	1.10–3.50	H = 11.298^*^	0.010^*^
Median (IQR)	2.06 (1.07–2.48)	2.55 (2.15–2.90)	2.0 (1.70–2.35)	1.80 (1.30–2.20)		
**vs. Control (p**_**0**_ **)**	0.888	0.003^*^	0.624			
**Pairwise**	p_1_=0.004^*^,p_2_ = 0.714,p_3_ = 0.016^*^					
**tRF-Gly-GCC**						
Min. – Max.	9.01–20.12	-3.34–17.63	-1.25–8.05	-13.07– 11.92	H = 54.416^*^	< 0.001^*^
Median (IQR)	13.0 (11.13–14.52)	9.15 (2.95–14.29)	1.02 (0.10–1.97)	-0.08 (-0.92–0.75)		
**vs. Control (p**_**0**_ **)**	< 0.001^*^	< 0.001^*^	0.251			
**Pairwise**	p_1_=0.054,p_2_ < 0.001^*^,p_3_ = 0.001^*^					

IQR: interquartile range SD: standard deviation U: Mann Whitney test.

H: H for Kruskal Wallis test, pairwise comparison between each 2 groups was done using post hoc test (Dunn’s for multiple comparisons test).

F: F for one way ANOVA test, pairwise comparison between each 2 groups was done using post hoc test (Tukey).

p_0_: p value for comparing between control and groups.

p_1_: p value for comparing between CRC and active CD.

p_2_: p value for comparing between CRC and inactive CD.

p_3_: p value for comparing between active and inactive CD.

*Statistically significant at *p* ≤ 0.05.

### Diagnostic performance of biomarkers

**Fig. 3:**
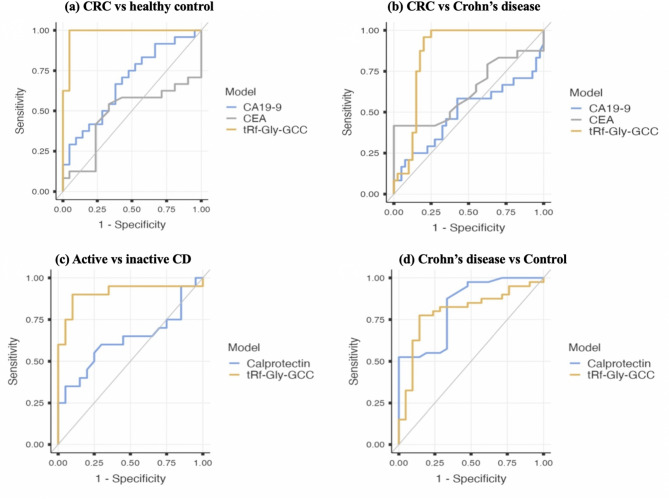
ROC curve analysis of serum tRF-Gly-GCC, fecal calprotectin, and conventional tumor markers across clinical cohorts. **(a)** CRC vs. healthy controls (*n* = 24 vs. *n* = 21): tRF-Gly-GCC (orange): AUC = 0.982, [95% CI: 0.946–1.000], cut-off > 9.006, sensitivity = 95.83% (95% CI 78.9–99.9), specificity = 95.2% (95% CI 76.2–99.9), *p* < 0.001. CA19-9 (blue): AUC = 0.679, [95% CI: 0.522–0.835], cut-off > 20, sensitivity = 66.7% (95% CI 44.7–84.4), specificity = 61.9% (95% CI 38.4–81.9), *p* = 0.041. CEA (grey): AUC = 0.482, [95% CI: 0.341–0.695], *p* = 0.838 (non-significant; no cut-off reported). tRF-Gly-GCC substantially outperforms both conventional markers for this comparison. **(b)** CRC vs. CD (*n* = 24 vs. *n* = 40): the CD reference group comprises all 40 CD patients (active and inactive combined). tRF-Gly-GCC (orange): AUC = 0.865, [95% CI: 0.769–0.960], cut-off > 9.71, sensitivity = 95.83% (95% CI 78.9–99.9), specificity = 80.0% (95% CI 64.4–90.9), *p* < 0.001. CEA (grey): AUC = 0.627, [95% CI: 0.470–0.783], *p* = 0.092 (non-significant). CA19-9 (blue): AUC = 0.498, [95% CI: 0.341–0.662], *p* = 0.983 (non-significant). No cut-off values are reported for non-significant markers. **(c)** Active vs. inactive Crohn’s disease (*n* = 20 vs. *n* = 20): tRF-Gly-GCC (orange): AUC = 0.910, [95% CI: 0.765–1.000], cut-off > 2.3, sensitivity = 90.0% (95% CI 68.3–98.8), specificity = 90.0% (95% CI 62.1–96.8), *p* < 0.001. Fecal calprotectin (blue): AUC = 0.629, [95% CI: 0.449–0.808], *p* = 0.164 (non-significant; no cut-off reported). tRF-Gly-GCC effectively differentiates Crohn’s disease activity, while fecal calprotectin did not reach statistical significance. **(d)** Crohn’s disease vs. healthy controls (*n* = 40 vs. *n* = 21): tRF-Gly-GCC (orange): AUC = 0.799, [95% CI: 0.688–0.924], cut-off > 0.93, sensitivity = 77.5% (95% CI 58.8–87.3), specificity = 85.7% (95% CI 69.6–98.8), *p* < 0.001. Fecal calprotectin (blue): AUC = 0.829, [95% CI: 0.721–0.937], cut-off > 43 mg/kg, sensitivity = 87.5% (95% CI 73.2–95.8), specificity = 66.7% (95% CI 43.0–85.4), *p* < 0.001.

ROC, receiver operating characteristic; AUC, area under the curve; CI, confidence interval; CRC, colorectal cancer; CD, Crohn’s disease; CA19-9, carbohydrate antigen 19 − 9; CEA, carcinoembryonic antigen; tRF-Gly-GCC, 5′-tRNA-derived fragment glycine GCC; sens, sensitivity; spec, specificity.

ROC analyses revealed strong diagnostic performance of tRF-Gly-GCC for distinguishing CRC patients from healthy controls (AUC = 0.982, 95% CI 0.946–1.000, *p* < 0.001). At a cut-off > 9.006, it achieved 95.8% sensitivity (95% CI 78.9–99.9) and 95.2% specificity (95% CI 76.2–99.9). Conventional markers CA19-9 and CEA demonstrated markedly inferior performance (AUC 0.679 and 0.482, respectively) (Table 3; Fig. 3a).

tRF-Gly-GCC distinguished the combined CD cohort (active and inactive disease) from CRC, achieving an AUC of 0.865 (95% CI 0.769–0.960, *p* < 0.001, cut-off > 9.71) with 95.8% sensitivity (95% CI 78.9–99.9) and 80.0% specificity (95% CI 64.4–90.9). The ROC comparison used all 40 CD patients (active and inactive combined) as the reference group. CEA (AUC 0.627, *p* = 0.092) and CA19-9 (AUC 0.498, *p* = 0.983) had no significant discriminatory value (Table 3; Fig. 3b).

tRF-Gly-GCC demonstrated the highest diagnostic accuracy for distinguishing active from inactive Crohn’s disease (AUC = 0.910, 95% CI 0.765–1.000, *p* < 0.001), with 90.0% sensitivity (95% CI 68.3–98.8) and 90.0% specificity (95% CI 62.1–96.8), whereas fecal calprotectin did not reach statistical significance (AUC = 0.629, *p* = 0.164) (Table 3; Fig. 3c).

When differentiating CD patients from healthy controls, tRF-Gly-GCC showed good diagnostic performance (AUC = 0.799, 95% CI 0.688–0.924, *p* < 0.001). At a cut-off of > 0.93, it achieved 77.5% sensitivity (95% CI 58.8–87.3) and 85.7% specificity (95% CI 69.6–98.8). Calprotectin showed a comparable AUC (0.829, *p* < 0.001) but with lower specificity (66.7%) at its optimal threshold, whereas tRF-Gly-GCC offered superior specificity (85.7%) for this comparison (Table 3; Fig. 3d).

Within the Crohn’s disease cohort, tRF-Gly-GCC correlated positively with CDAI (r*s* = 0.523, *p* = 0.001) and SES-CD (r*s* = 0.645, *p* < 0.001), but showed no significant correlation with fecal calprotectin (r*s* = 0.204, *p* = 0.207) (Table 3).

**Table 3:** Diagnostic performance of serum tRF-Gly-GCC and conventional markers across disease groups (Section A) and Spearman rank correlations within the Crohn’s disease cohort (Section B).

A. ROC Diagnostic Performance											
Comparison	Marker		AUC	p	95% C.I		Cut off^#^	Sensitivity % (95% CI)	Specificity % (95% CI)	PPV	NPV
Control vs. CRC	CA19-9		0.679	0.041^*^	0.522–0.835		> 20^a^	66.67	61.9	66.7	61.9
	CEA		0.482	0.838	0.341–0.695			(44.7–84.4)	(38.4–81.9)		
	tRF-Gly-GCC		0.982	< 0.001^*^	0.946–1.000		> 9.006	95.83 (78.9–99.9)	95.2 (76.2–99.9)	95.8	95.2
CD vs. CRC	CA19-9		0.498	0.983	0.341–0.662						
	CEA		0.627	0.092	0.470–0.783						
	tRF-Gly-GCC		0.865	< 0.001^*^	0.769–0.960		> 9.71	95.83 (78.9–99.9)	80.0 (64.4–90.9)	74.2	97.0
Inactive vs. active CD	tRF-Gly-GCC		0.910	< 0.001^*^	0.765–1.000		> 2.3	90.0 (68.3–98.8)	90.0 (62.1–96.8)	90.0	90.0
	Calprotectin		0.629	0.164	0.449–0.808						
Control vs. CD	tRF-Gly-GCC		0.799	< 0.001^*^	0.688–0.924		> 0.93	77.5 (58.8–87.3)	85.7 (69.6–98.8)	93.7	65.5
	Calprotectin		0.829	< 0.001^*^	0.721–0.937		> 43	87.50 (73.2–95.8)	66.67 (43.0-85.4)	83.3	73.7

B. Spearman correlation of tRF-Gly-GCC within CD Cohort (*n* = 40)		
Parameter	r_s_	p
Calprotectin	0.204	0.207
CDAI score	0.523^*^	0.001^*^
SES–CD score	0.645^*^	< 0.001^*^

AUC: Area under the curve, p value: probability value, CI: confidence intervals, NPV: negative predictive value PPV: positive predictive value, r_s_: Spearman coefficient.

*Statistically significant at *p* ≤ 0.05.

^a^Cut off was chosen according to Youden index.

### Confounding analysis and multivariable regression

**Fig. 4:**
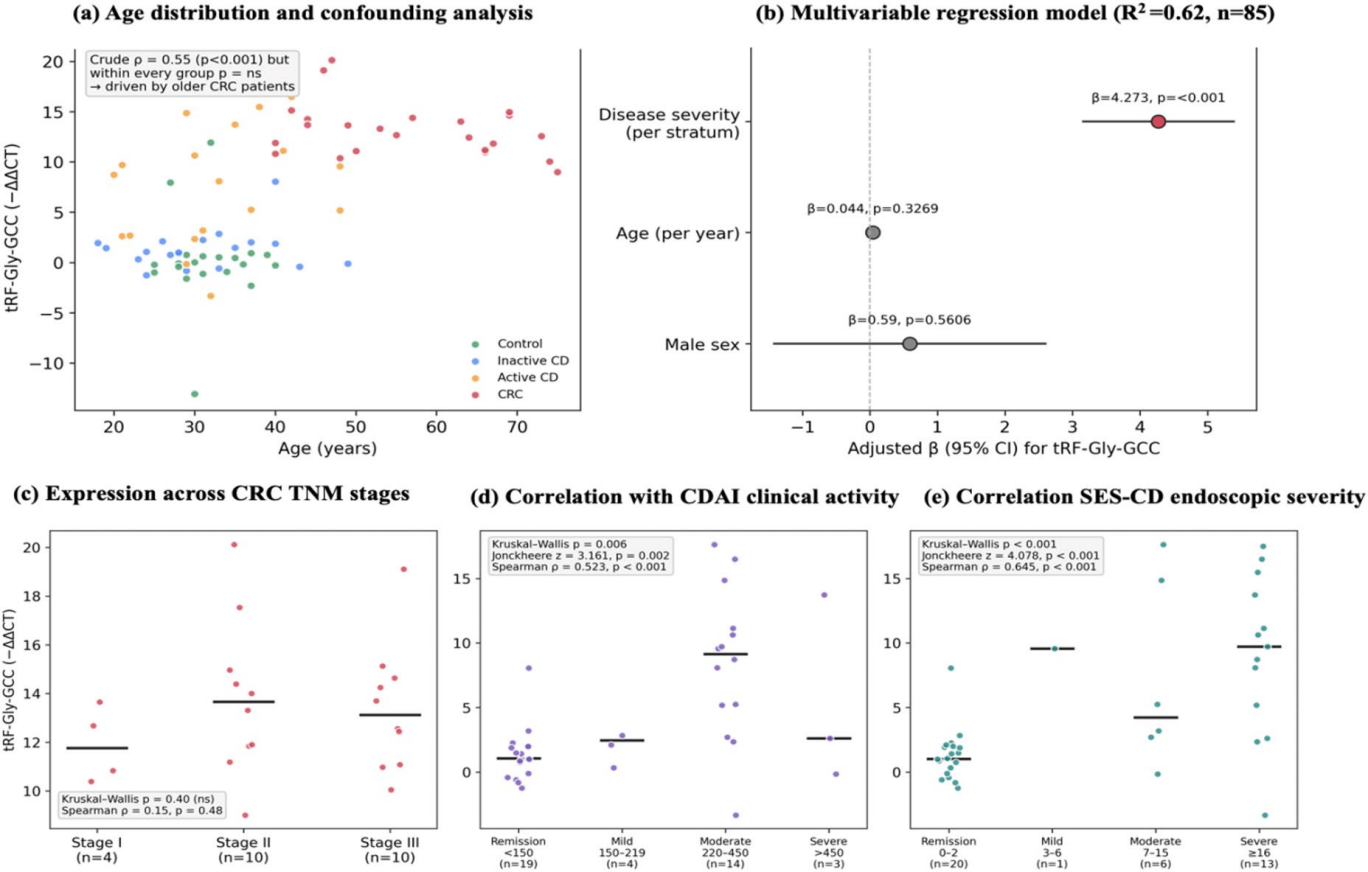
Clinical determinants and multivariable assessment of serum tRF-Gly-GCC expression. **(a)** Age distribution and confounding analysis. Stratified scatter plot of tRF-Gly-GCC levels versus age across clinical cohorts. Relative abundance of the tRNA-derived fragment tRF-Gly-GCC is quantified on the y-axis using the -ΔΔCT method (where higher values denote increased expression). Individual patients are color-coded by clinical group: Healthy Control (green), Inactive Crohn’s Disease (CD; blue), Active CD (orange), and Colorectal Cancer (CRC; red). Although a crude univariable analysis indicates a significant positive correlation between tRF-Gly-GCC levels and age across the entire cohort (Spearman’s r_s_ = 0.55, *p* < 0.001), this relationship is non-significant within every individual clinical stratum, indicating a confounding effect driven by the advanced age of the CRC cohort. **(b)** Multivariable regression model adjusting for demographic variables. Forest plot from the ordinal parameterisation of the multivariable linear regression model (disease stratum coded 1–4 as a continuous predictor; *n* = 85; R² = 0.622, Adjusted R² = 0.608). Disease stratum is the only significant predictor (β = 4.27 per stratum unit, 95% CI 3.16–5.39, *p* < 0.001); age (β = 0.044, *p* = 0.327) and sex (β = 0.59, *p* = 0.561) are non-significant. Group-specific coefficients from the dummy-coded parameterisation are provided in Table 4. **(c)** Expression across CRC TNM stages. Subgroup analysis of the colorectal cancer cohort revealing that tRF-Gly-GCC expression does not significantly vary across advancing TNM tumor stages (Kruskal–Wallis *p* = 0.40; Jonckheere–Terpstra *p* = 0.48; Spearman r_s_ = 0.15, *p* = 0.485). **(d)** Correlation with CDAI clinical activity. Within the Crohn’s disease cohort, tRF-Gly-GCC expression shows a significant overall increase across CDAI clinical-activity categories (Jonckheere–Terpstra z = 3.161, *p* = 0.002; Spearman r_s_ = 0.523, *p* < 0.001), consistent with worsening inflammatory disease, though the small severe subgroup (*n* = 3) showed anomalously low values. **(e)** Correlation with SES-CD endoscopic severity. Biomarker expression similarly exhibits a highly significant monotonic increase correlating with objective endoscopic mucosal damage (SES-CD) (Jonckheere-Terpstra z = 4.078, *p* < 0.001; Spearman’s r_s_ = 0.645, *p* < 0.001). Solid black lines represent subgroup medians. Subgroup analysis by CRC TNM stage, CDAI, and SES-CD scores are provided in Supplementary Table S3. tRF, tRNA-derived fragment; CT, cycle threshold; CI, confidence interval; CRC, colorectal cancer; CD, Crohn’s disease; TNM, tumor-node-metastasis; CDAI, Crohn’s Disease Activity Index; SES-CD, Simple Endoscopic Score for Crohn’s Disease.

The influence of demographic and clinical variables on tRF-Gly-GCC expression was assessed using univariate analyses and multivariable regression models (Table 4; Fig. 4a–b). In univariate analysis, tRF-Gly-GCC showed a positive correlation with age in the overall cohort (Spearman rs = 0.55, *p* < 0.001), although this association was not consistently observed within individual disease strata. No association with sex was detected (*p* = 0.10), and disease duration was not correlated with expression in the Crohn’s disease subgroup (rs = − 0.01, *p* = 0.96).

In multivariable analysis of the full cohort (tRF ~ disease stratum + age + sex; R² = 0.622, adjusted R² = 0.608; *n* = 85), disease stratum was the only significant predictor of tRF-Gly-GCC expression (β = 4.27 per stratum unit, 95% CI 3.16–5.39, *p* < 0.001), whereas age and sex were not independently associated. The ordinal parameterisation is shown in Fig. 4b.

Consistent with this, the categorical model showed significantly higher expression in active Crohn’s disease (β = 8.31, *p* < 0.001) and colorectal cancer (β = 12.74, *p* < 0.001) compared with healthy controls, while inactive Crohn’s disease was not significantly different (β = 1.12, *p* = 0.390), with a slight improvement in model fit (R² = 0.654, adjusted R² = 0.632 ). (Table 4) Within the Crohn’s disease cohort (tRF ~ SES-CD + age + sex + disease duration; R² = 0.464, adjusted R² = 0.385; *n* = 40), endoscopic severity was the only significant predictor (β = 0.28 per SES-CD point, 95% CI 0.14–0.43, *p* < 0.001), while age, sex, and disease duration were not significant.

**Table 4:** Multivariable linear regression for serum tRF-Gly-GCC: independence from demographic covariates.

tRF-Gly-GCC	β	95% CI	*p*
**Groups**			
CRC	12.742	9.030–16.453	< 0.001*
Active CD	8.311	5.664–10.958	< 0.001*
Inactive CD	1.124	-1.463–3.710	**0.390**
Control	**0.000**	**0.000**	
**Age (years)**	0.014	-0.093–0.122	**0.792**
**Sex**	0.59	-1.42–2.60	**0.56**
Male	0.235	-1.734–2.205	**0.813**
Female	**0.000**	**0.000**	
R^2^ = 0.654 (adjusted R^2^ = 0.632)			
CD cohort (*n* = 40)			
SES-CD (per point)	0.28	0.14–0.43	< 0.001*
Age (years)	0.17	-0.02–0.36	0.083
Sex (Male)	1.84	-1.42–5.09	0.26
Disease duration (years)	−0.00	−0.39–0.39	0.995

R^2^ = 0.464 (adjusted R^2^ = 0.385).

β, unstandardised regression coefficient; CI, confidence interval; R², coefficient of determination; SES-CD, simple endoscopic score for Crohn’s disease; CD, Crohn’s disease.

*Statistically significant at *p* ≤ 0.05.

### Subgroup analyses by disease severity

In the CRC cohort, tRF-Gly-GCC expression did not differ significantly across TNM stages I–III (Kruskal–Wallis *p* = 0.40; Jonckheere–Terpstra *p* = 0.48; Spearman r_s_ = 0.15, *p* = 0.485). Expression was already markedly elevated in Stage I disease (median 11.76) and remained consistently high in Stages II and III (medians 13.66 and 13.13, respectively). However, only four patients were classified as Stage I, and conclusions regarding early-stage disease should therefore be interpreted cautiously.

In the CD cohort, tRF-Gly-GCC expression increased across CDAI clinical-activity categories (Kruskal–Wallis *p* = 0.006; Jonckheere–Terpstra z = 3.161, *p* = 0.002; Spearman r_s_ = 0.523, *p* < 0.001), with median values rising from remission (1.05) to mild (2.47) and moderate disease (9.15). The severe CDAI subgroup (*n* = 3, median 2.60) did not exhibit the highest expression levels. The relationship with SES-CD was stronger and more consistently monotonic, with tRF-Gly-GCC levels increasing significantly across endoscopic severity categories (Kruskal–Wallis *p* < 0.001; Jonckheere–Terpstra z = 4.078, *p* < 0.001; Spearman r_s_ = 0.645, *p* < 0.001), from endoscopic remission (median 1.02) to severe disease (median 9.71). Subgroup analyses stratified by CRC TNM stage, CDAI clinical-activity category, and SES-CD endoscopic-severity category are presented in Supplementary Table S3 and Fig. 4 (panels c–e).

## Discussion

Chronic intestinal inflammation is a feature of both IBD and sporadic colorectal cancer^47,48^. In IBD, repeated mucosal injury is linked to DNA damage and epigenetic changes^49^. In sporadic CRC, both genetic and non-genetic factors, including hereditary variants, family history, lifestyle, and environmental exposures, contribute to low-level intestinal inflammation^4,50^. In both conditions, the intestinal microenvironment shows signs of ongoing inflammation^3^. These observations highlight the value of molecular markers that reflect inflammatory activity in intestinal disease^4,47^. Non-coding RNAs and tRNA-derived fragments have emerged as important regulators of cellular homeostasis, immunological signalling, and neoplastic transformation^27,32,51,52^.

In the present study, circulating tRF-Gly-GCC levels showed a directional increase across disease states, moving from healthy controls to inactive CD, active CD, reaching their highest values in CRC. This ordered pattern was formally confirmed by a Jonckheere–Terpstra test for ordered alternatives. While the principal separation occurred between quiescent states (healthy controls and inactive CD) and active or neoplastic states (active CD and CRC), it is important to note that two adjacent pairwise comparisons, specifically healthy controls versus inactive CD and active CD versus CRC, did not reach statistical significance. Nevertheless, the overall ordered trend remains significant.

The lack of distinction between healthy controls and inactive CD is consistent with the quiescent inflammatory state of inactive disease. Furthermore, the molecular overlap between active CD and CRC likely reflects shared molecular features associated with sustained inflammation, tissue stress, and dysregulated gene expression programs. Both conditions are characterized by substantial cellular stress, inflammatory signaling, tissue remodeling, and dysregulated gene expression. Indeed, previous experimental studies have shown that tRF-Gly-GCC expression can be induced under stress conditions, including endoplasmic reticulum stress, and that this fragment may be associated with increased cell proliferation, migration, and oncogenic signaling^34,36,53^. Our findings are consistent with the concept that chronic inflammation increases tRF-Gly-GCC expression, potentially contributing to a molecular environment conducive to malignant transformation. Consequently, we emphasize that tRF-Gly-GCC reflects increasing molecular perturbation across disease states rather than implying direct biological progression from CD to CRC.

Our results for tRF-Gly-GCC are in line with Wu et al.^34^, who found higher circulating 5′-tRF-Gly-GCC levels in colorectal cancer patients, suggesting its potential as a diagnostic marker. Wan et al.^54^ recently confirmed upregulation of 5′-tRF-Gly-GCC in colorectal cancer tissues and serum, with functional assays suggesting a pro-tumorigenic role. Similarly, Xu et al.^55^ showed that the 5′ half fragment tsRNA-Gly-GCC acts as an oncogenic molecule, promoting resistance to 5-fluorouracil by activating the JAK1/STAT6 pathway. In contrast, Christodoulou et al.^56^ reported lower levels of tRF-Gly-GCC within tumor tissue compared with adjacent normal tissue, where higher expression was linked to poorer survival. This might suggest that its effects may depend on fragment subtype or cellular localization. To our knowledge, no studies have evaluated circulating tRF-Gly-GCC in inflammatory bowel disease, highlighting the novelty of our findings and extending the relevance of this biomarker beyond malignancy to encompass chronic inflammatory disease.

Within the CRC cohort, tRF-Gly-GCC expression was elevated across all TNM stages, including early-stage disease, and showed no evidence of a stage-dependent pattern. This sustained elevation is compatible with the possibility that dysregulation of tRF-Gly-GCC may occur relatively early in colorectal tumorigenesis and persist throughout disease progression rather than increasing with advancing stage. This pattern is consistent with its proposed involvement in stress-induced oncogenic signalling^34,53^. The absence of a stage-related gradient should, however, be interpreted cautiously, as metastatic disease was not represented in the cohort and may exhibit distinct molecular characteristics, while the limited number of early-stage cases may have reduced the ability to detect subtle stage-related differences.

Among patients with CD, tRF-Gly-GCC correlated positively with CDAI and SES-CD, indicating a stronger relationship with clinical and endoscopic disease activity. Subgroup analyses demonstrated that the association with endoscopic severity (SES-CD) was stronger and more consistently monotonic than with CDAI categories, suggesting that tRF-Gly-GCC more closely tracks objective mucosal inflammatory burden than symptom-based measures. The anomalous severe CDAI subgroup (*n* = 3) that did not exhibit the highest tRF-Gly-GCC expression likely reflects the known susceptibility of CDAI to non-inflammatory symptoms. These findings support tRF-Gly-GCC as an objective molecular complement to endoscopic assessment of mucosal inflammatory activity.

In contrast, no significant correlation was observed between tRF-Gly-GCC expression and fecal calprotectin levels, suggesting that this fragment may reflect molecular stress or regulatory activity not detected by conventional markers of intestinal inflammation. Although fecal calprotectin is widely used as a non-invasive marker of intestinal inflammation, its levels are influenced by endoscopic lesion characteristics. In a prospective cohort, fecal calprotectin increased significantly with the presence and depth of ulcerations and the affected mucosal surface in CD, indicating greater release with more severe endoscopic damage. Furthermore, diagnostic studies have shown that the ability of fecal calprotectin to discriminate active from inactive Crohn’s disease varies depending on clinical and disease characteristics. Assay thresholds and patient subgroup features can influence sensitivity and specificity, limiting its performance in certain populations^57,58^.

Conventional inflammatory and tumor markers were elevated in disease states but lacked the specificity needed to differentiate inflammation from malignancy. In contrast, tRF-Gly-GCC effectively distinguished active from inactive Crohn’s disease and colorectal cancer from CD, suggesting responsiveness to inflammatory activity as well as oncogenic changes. Overall, tRF-Gly-GCC outperformed traditional markers in differentiating disease states.

Multivariable adjustment showed that the association between tRF-Gly-GCC and disease state remained statistically significant after adjustment for age and sex. The crude age correlation observed across the full cohort was attributable to the older age distribution of the CRC group; within each individual group no significant within-group age association was found. Similarly, sex and disease duration did not independently predict tRF-Gly-GCC expression. The CRC age difference is expected given the epidemiology of sporadic colorectal cancer and does not confound the biomarker associations.

In conclusion, circulating tRF-Gly-GCC may serve as a valuable blood marker in both chronic intestinal inflammation and colorectal cancer. Its directional rise from healthy controls through inactive and active Crohn’s disease to colorectal cancer suggests a role in both inflammatory activity and tumor development. Further long-term and experimental studies are needed to confirm its clinical significance and to better define its biological functions in these disease contexts.

This study has several limitations. The cross-sectional design limited the evaluation of changes in biomarker expression over time. Longitudinal investigations are warranted to determine whether tRF-Gly-GCC can serve as a predictor of progression from CD to colorectal cancer. Crohn’s disease was selected as the model due to the limited research exploring its association with cancer development. Including ulcerative colitis in future studies could offer valuable comparative insights and contribute to a broader understanding of inflammation-driven carcinogenesis. Detailed medication records were unavailable, and treatment exposure, therefore, remains a potential confounder, warranting future prospective investigation. In addition, subgroup analyses were limited by small and uneven sample sizes across CRC stages and Crohn’s disease activity groups, which may have reduced statistical power to detect subtle group differences and limits characterization of biomarker behavior across disease severity. Furthermore, our observed expression ranges and diagnostic cut-offs require validation in larger, balanced cohorts covering all CRC stages and inflammatory states to confirm these findings and better clarify the clinical utility of tRF-Gly-GCC.

Finally, the molecular overlap between active Crohn’s disease and colorectal cancer may reduce the biomarker’s discriminatory performance at this specific disease boundary. This highlights the need for further functional analyses to clarify how inflammatory processes influence RNA expression and to verify the involvement of these molecules in cancer progression.

## Supplementary Information

Below is the link to the electronic supplementary material.

Supplementary Material 1

## Data Availability

The datasets used during the current study are available from the corresponding author on reasonable request, subject to compliance with ethical and privacy restrictions regarding participant data.
