# Supplementary material for: Circulating tRF-Gly-GCC as a biomarker for colorectal cancer and Crohn’s disease activity
Source: Sci Rep. 2026 Jun 30;16:19941. doi: 10.1038/s41598-026-59591-8 (PMC13319805; doi:10.1038/s41598-026-59591-8)
Supplement: Supplementary file 1 — Supplementary Material 1 [file 41598_2026_59591_MOESM1_ESM.docx]

**Supplementary**

**Supplementary Table S1 Primer set designed for amplification of cDNA for tRF-Gly-GCC and U6 gene sequences.**

| **Primer** | **Sequence** **(5'-3')** | **Tm (^◦^C)** |
| --- | --- | --- |
| **tRF-Gly-GCC gene** |  | |
| Forward primer | CATTGGTGGTTCAGTGGTAGAAT | 55°C |
| Reverse primer | AGTGCAGGGTCCGAGGTATT | 55°C |
| **U6 gene** |  | |
| Forward primer | CTCGCTTCGGCAGCACA | 55°C |
| Reverse primer | AACGCTTCACGAATTTGCGT | 55°C |

*Tm, melting temperature*

**Supplementary Table S2. Reference Intervals and Jonckheere–Terpstra Trend Analysis of Relative Expression Among Ordered Disease Groups**

| **Group (ordered)** | **n** | **Median (−ΔΔCT)** | **95% CI of Median** | **IQR** | **10th–90th Centile** |
| --- | --- | --- | --- | --- | --- |
| Healthy control | 21 | −0.08 | −0.43 to 0.62 | −0.92 to 0.75 | −1.61 to 0.93 |
| Inactive CD | 20 | 1.02 | 0.54 to 1.91 | 0.21 to 1.95 | −0.61 to 2.32 |
| Active CD | 20 | 9.15 | 4.19 to 12.43 | 3.07 to 14.00 | 2.09 to 16.60 |
| CRC | 24 | 13.00 | 11.84 to 14.25 | 11.16 to 14.46 | 10.52 to 16.82 |
| **Kruskal-Wallis** | | H = 54.416, p < 0.001 | | | |
| **Jonckheere-Terpstra** | | z = 7.896, p < 0.001 | | | |
| **Spearman (ordinal severity)** | | r_s_ = 0.793, p < 0.001 | | | |

*CI, confidence interval; IQR, interquartile range; JT, Jonckheere–Terpstra test; Z, standardized test statistic;*

*r_s_, Spearman's rank correlation coefficient; p, probability*

**: statistically significant at p ≤ 0.05.*

**Supplementary Table S3. Subgroup analysis: serum tRF-Gly-GCC expression by CRC TNM stage, CDAI clinical-activity category, and SES-CD endoscopic-severity category.**

| **Stratification / Subgroup** | **n** | **Median tRF (−ΔΔCT)** | **IQR** | **KW p** | **J-T z (p)** | **Spearman ρ (p)** |
| --- | --- | --- | --- | --- | --- | --- |
| **CRC — TNM Stage Subgroups (overall: KW p = 0.40; J-T p = 0.48; ρ = 0.15, p = 0.485)** | | | | | | |
| Stage I | 4 | 11.76 | 10.72 – 12.92 | 0.40 | 0.48 (NS) | 0.15 (0.485) |
| Stage II | 10 | 13.66 | 11.86 – 14.83 |  |  |  |
| Stage III | 10 | 13.13 | 11.43 – 14.54 |  |  |  |
| **CD — CDAI Clinical Activity (overall: KW p = 0.006; J-T z = 3.161, p = 0.002; ρ = 0.523, p < 0.001)** | | | | | | |
| Remission (< 150) | 19 | 1.05 | 0.32 – 1.97 | 0.006 | z = 3.161 (0.002) | 0.523 (< 0.001) |
| Mild (150 – 219) | 4 | 2.47 | 1.66 – 6.00 |  |  |  |
| Moderate (220 – 450) | 14 | 9.15 | 5.19 – 11.01 |  |  |  |
| Severe (> 450)§ | 3 | 2.60 | 1.23 – 8.16 |  |  |  |
| **CD — SES-CD Endoscopic Severity (overall: KW p < 0.001; J-T z = 4.078, p < 0.001; ρ = 0.645, p < 0.001)** | | | | | | |
| Remission (0 – 2) | 20 | 1.02 | 0.21 – 1.95 | < 0.001 | z = 4.078 (< 0.001) | 0.645 (< 0.001) |
| Mild (3 – 6) | 1 | 9.57 | — |  |  |  |
| Moderate (7 – 15) | 6 | 4.23 | 2.82 – 12.45 |  |  |  |
| Severe (≥ 16) | 13 | 9.71 | 5.17 – 13.72 |  |  |  |

*KW, Kruskal–Wallis; J–T, Jonckheere–Terpstra ordered-trend test;*

*ρ, Spearman correlation with the underlying continuous score.*

**: statistically significant at p ≤ 0.05*
